# An association between the physical activity level and skeletal muscle mass index in female university students with a past exercise habituation

**DOI:** 10.1016/j.afos.2021.10.002

**Published:** 2021-10-26

**Authors:** Kazushige Oshita, Ryota Myotsuzono

**Affiliations:** a Department of Human Information Engineering, Okayama Prefectural University, 111 Kuboki, Soja-shi, Okayama, 719-1197, Japan; b Department of Sports Science, Kyushu Kyoritsu University, 1-8, Jiyugaoka, Yahatanishi-ku, Kitakyushu-shi, Fukuoka, 807-8585, Japan

**Keywords:** Body composition, Leg muscle mass, Appendicular muscle mass, Lean body mass, Sarcopenia

## Abstract

**Objectives:**

This study aims to investigate the association between skeletal muscle mass index (SMI) and physical activity among female university students who had exercise habituation in junior and high school.

**Methods:**

The body composition of 120 Japanese female students was measured using the bioelectrical impedance analysis (BIA) method, and their physical activity level (PAL) was measured using a factorial method. Based on the ‘Dietary Reference Intakes for Japanese’ (DRIs-J), according to the Ministry of Health, Labour and Welfare, PAL (24-h energy consumption/basal metabolic rate) classifications were defined as low-PAL (PAL < 1.6), moderate-PAL (1.6 ≤ PAL < 1.9), and high-PAL (1.9 ≤ PAL < 2.2), respectively.

**Results:**

Individuals with low-PAL had a significantly lower SMI, especially for the lower limb muscles, than individuals with moderate-PAL or higher. More than 50% of the individuals with currently low-PAL corresponded or tended to correspond to the SMI cut-off value defined by the Asian Working Group for Sarcopenia or the 2017 National Health and Nutrition Survey of Japan. Therefore, more than half of the female students with currently low-PAL, even those with an exercise habituation in the past, corresponded to the cut-off value for muscle loss in sarcopenia diagnosis, particularly in the lower limbs.

**Conclusions:**

These results suggest that it is important to maintain a moderate or higher level of physical activity in the DRIs-J classification, even for young women who used to exercise in the past, to maintain muscle mass accordingly.

## 1 Introduction

Age-related loss of skeletal muscle mass and function in the older population is described as ‘sarcopenia’ [1], and it is significantly and independently associated with functional impairment and disability [2]. Although age, sex, body size, level of physical activity (PA), and heredity are considered to be factors affecting sarcopenia, the importance of PA and resistance exercise training is widely recognized as the most effective intervention to slow down the decline in muscle mass and strength [3]. Nevertheless, there remains considerable unexplained variability in the age-related decline in muscle mass or strength, suggesting that peak values achieved in early adulthood may be an explanatory factor in addition to the effects of aging [3,4]. Therefore, most of the research on sarcopenia to date has focused on the modification of age-related decline, however, the effects of peak muscle mass and strength achieved in early adulthood have also been indicated in some studies [[3], [4], [5]]. In particular, several studies have reported that the rate of loss of muscle mass with aging is greater in females than in males [6], [7], suggesting therefore, that the acquisition of peak values in early adulthood may be more important in females.

To diagnose sarcopenia, muscle mass is evaluated by the skeletal muscle mass index (SMI), which is calculated by dividing the sum of the appendicular muscle mass (AMM) in kilograms by the square of the body height in meters [8,9]. The European [8] and Asian Working Group for Sarcopenia (AWGS) [9] have defined the SMI cut-off value as 2 standard deviations (SD) below the mean of the young population. Although sarcopenia is diagnosed at the age of 65 years or older, some studies have evaluated SMI or AMM in a young female population to prevent sarcopenia in the future [5,10,11]. With regard to PA, it has been reported that both the current level of PA and exercise habituation at 16–18 years of age are significant predictors of SMI and AMM in women in early adulthood [5]. This study suggests that past exercise habituation and current PA levels are important for achieving peak SMI in early adulthood. On the other hand, a systematic review of PA in collegiate students revealed that PA in freshmen decreased compared to when they were in high school [12]. Further, Butler et al. [13] reported that, compared to their levels upon entry into college, leisure, sports, and occupational activities, as well as lean body mass (LBM), decreased significantly (with an increase in body fat) in freshman females 5 months after they entered the university. From these previous studies, it can be concluded that some of the female university students, with a low level of PA currently, may also have lower SMI, even if they had an exercise habituation prior to their gaining an entry in the university.

PA is classified and evaluated into several levels by various organizations, including the Food and Agriculture Organization of the United Nations/World Health Organization/United Nations University (FAO/WHO/UNU) expert consultation on human energy requirements [14], the Institute of Medicine (IOM) -National Academy of Medicine [15], and the dietary reference intakes for Japanese (DRIs-J) [16]. As mentioned above, exercise habituation in the past is an important factor that predicts SMI in young women [5]. However, when PA decreases due to lifestyle changes at university, SMI may decrease even in those individuals who had a habit of exercising in the past. In particular, it may be of greater importance to focus on changes in muscle mass (ie, SMI) with changes in PA, since it has been reported that muscle strength is maintained or declines slower while muscle mass declines with detraining [17]. Furthermore, exercise interventions in older adults may have a role in improving muscle strength and physical performance, but not muscle mass [18]. Therefore, the acquisition of peak muscle mass in early adulthood will be a more important factor in preventing sarcopenia in the future than muscle strength. If research is conducted to study the association between current PA classification and SMI in young women with exercise habituation in the past, its findings can be used to suggest a certain PA level (PAL) that can help prevent muscle loss in early adulthood. However, the relationship between the current PAL and SMI in young females who used to exercise in the past is not known. Therefore, the present study was performed to investigate the association between current PAL, SMI, and AMM in female university students who exercised when they were in junior and high schools.

## 2 Methods

### 2.1 Participants

Two hundred Japanese female university students (aged 18–21 years) were included in the study. Participants were asked about the athletic clubs they belonged to in junior and high schools in order to focus on female students with a past exercise habituation in this study, and those participants who were not in athletic clubs or were non-athletic members of these clubs were excluded accordingly. Furthermore, since an extremely high PAL affects SMI, the participants who had PAL (24-hour energy consumption/basal metabolic rate) outside the range described in DRIs-J [16] (described later), that is, PAL ≥ 2.2, were also excluded. Therefore, finally the remaining 120 participants were included in the present study.

All participants were informed verbally and in writing beforehand that their responses to the questionnaire would be anonymous and used only for research purposes. They were also informed that the obtained data would be published after statistical analysis in such a way that the individuals could not be identified. The survey was conducted only when these terms were accepted by the participants. This study was approved by the ethics committee of Kyushu Kyoritsu University (No. 2018-15).

The breakdown of the proportions of the participants involved in club activities during junior school was as follows: less than 5% for handball, kendo, badminton, gymnastics, soccer, and soft tennis, 5%–15% for softball, swimming, and athletics, 23.7% for volleyball, 28.8% for basketball, and 14.0% for others (including tennis, judo, etc.). The breakdown of the proportions of the participants involved in club activities during high school was as follows: less than 5% for badminton, gymnastics, soccer, kendo, table tennis, and softball, 5%–15% for swimming handball and athletics, 21.2% for volleyball, 26.3% for basketball, and 13.0% for others (including tennis, judo, aerobics dance, etc.).

### 2.2 Daily activity

In the daily activity survey, participants were asked about their average daily lives on weekdays for a month. Participants completed a questionnaire-based survey in which a day (24 hour) was divided into 288 periods of 5 minutes each. Activity for each 5-minute period was further divided into 8 categories.

PAL and SMI were assessed mainly according to the DRIs-J [16] and National Health and Nutrition Survey of Japan (NHNS-J) [19], since the participants of this study were Japanese. In DRIs-J [16], activities are classified into 5 categories: (1) sleeping, (2) static activity in sitting or standing, (3) low-intensity activities such as slow walking and housework; (4) moderate-intensity activities such as exercise/work that can be continued for a long period, for example: normal speed walking; and (5) high-intensity activities, such as exercise and work that frequently require rest after performing them for some time. In the present study, these categories were partially modified to correspond to the daily lives of university students. The activities from the categories (1) sleeping to (3) low-intensity activities were divided into 5 categories: sleep, non-sleep lying, sitting, standing, and low intensity activity. Furthermore, the exercise/sports activity time was separated from the original categories (4) moderate- and (5) high-intensity activities. Therefore, 8 categories in the present study were as follows: (A) sleeping; (B) lying down (excluding sleeping, eg, relaxing, etc.), (C) sitting (taking classroom lessons, reading books, watching TV, eating, etc.); (D) light work while standing (discourse, cooking, washing dishes, dressing, showering, etc.); (E) slow movements and housework (walking slowly, light cleaning, washing, etc.); (F) household chores/work that can be continued for a certain amount of time (normal walking, cycling, floor cleaning, preparation of futon, etc.); (G) high-intensity housework/labor that requires frequent resting (walking/cycling uphill, carrying heavy items, drying clothes); and (H) exercise/sports activities. Furthermore, participants were also asked about the details of their exercise and sports time. This was also divided into the following categories based on the average intensity of the activity: (a) resting time while standing or sitting (not including walking); (b) low-intensity exercise/sports (walking, biking, light exercise, light resistance training, etc.); (c) low-to moderate-intensity exercise/sports (tennis, gymnastics/dance, aqua aerobics, sports activity with long breaks such as baseball, golf, etc.); (d) moderate-intensity exercise/sports (long-distance running, resistance exercises, sports that repeat running and rest eg, soccer, etc.); and (e) high-intensity exercise/sports (high-intensity aerobics dance, basketball, sprint running, swimming, etc.).

The daily activity survey and the details of the exercise/sports activities were recorded using the collective survey method. The survey was conducted along with an explanation provided by a person who had expert knowledge of exercise and nutrition to enhance the credibility of the answers.

### 2.3 Estimation of PA-level by factorial method

The intensity of each activity was evaluated using an index that was expressed as a multiple of the basal metabolism (PA ratio; PAR = energy consumption/resting metabolic rate). According to DRIs-J [16], the value for sleeping was 1.0, 1.5 (1.1–1.9) for static activity such as sitting or standing, 2.5 (2.0–2.9) for low-intensity activities, 4.5 (3.0–5.9) for moderate-intensity activities, and 7.0 (6.0 or above) for high-intensity activities. The values from the FAO/WHO/UNU report [14,20] were also added (the figures in parentheses are based on the PAR for females from FAO/WHO/UNU [14,20]) to these values. PAR for each categorized activity was as follows: 1.0 for sleeping, 1.1 for lying down, 1.5 for sitting (1.3 for reading and office worker filing, 1.4 for writing, 1.8 for typing, 1.6 for eating, 1.72 for watching TV), 1.7 for light work like standing (1.5 for standing, 1.7 for dish washing, 1.8 for cooking), 2.5 for slow movements and housework (2.5 for walking, 2.7 for light cleaning, 2.8 for doing laundry), 4.0 for household chores/work that can be continued for a certain amount of time (3.5 for carrying 15–20 kg, 3.5 for cycling, 4.4 for cleaning floors, 3.4 for bed-making in summer), and 5.0 for high intensity household work/labor requiring frequent resting (5.4 for walking uphill, 4.9 for winter bed-making, 4.4 for hanging laundry outside).

PAR for exercise/sports activities were as follows: 1.7 for breaking time, 4.0 for low-intensity exercise/sports (3.6 for bicycling, 4.24 for low-intensity aerobic dance, 2.1–4.2 for light recreational activities like bowling, golfing, etc.), 5.0 for low-medium intensity exercise/sports (5.09 for tennis, 5.09 for dance, 4.2–6.3 for moderate level recreational activities like dancing, tennis, etc.), 6.5 for moderate-intensity exercise/sports (6.55 for long-distance running, 6.06 for volleyball, 6.29 for circuit training, 6.6 or higher for high-intensity recreational activities (soccer, jogging, etc.), and 8.0 for high-intensity exercise/sports (8.31 for high-intensity aerobic dance, 7.74 for basketball, 8.28 for sprint running, and 9.0 for swimming [The value for swimming is for males, as there was no value available for females]).

PAL was evaluated by multiplying each activity's duration and its corresponding PAR and calculating the average for one day. The total time spent lying down and sitting was calculated to arrive at the total sedentary time per day.

### 2.4 Physical characteristics

After the daily activity survey, height, weight, percentage of body fat, and AMM were measured and recorded for each participant using a daily survey questionnaire. AWGS provides a SMI cut-off value using the bioelectrical impedance analysis (BIA) method for the evaluation of muscle mass [9,21] (although the BIA method evaluates the lean appendicular mass, it is expressed as ‘AMM’ in the present study). Body composition was measured by the BIA method using a dual-frequency body composition analyzer (RD-E04, Tanita Co., Ltd., Tokyo, Japan) [11]. For an explanation of the brief principle of the BIA method, skeletal muscle contains a large volume of water, while body fat contains almost no water. Therefore, the BIA method estimates lean body mass and body fat mass based on the difference in electrical impedance of these tissues by flowing electric current in the body. In this study, the impedance values were measured from the right arm, left arm, right leg, left leg, and trunk by placing 2 electrodes each on the right and left palms and soles of the feet (8 electrodes in total) and by allowing 2 different frequencies of electric currents to flow through the body to estimate the composition of each part.

From each value, BMI was calculated by dividing body weight (kg) by height (m) squared. SMI was calculated by dividing the AMM (kg) by height (m) squared. The upper limb muscle mass (kg) was calculated as the total value of the left and right upper limb muscle masses, and the lower limb muscle mass was calculated as the total value of the left and right lower limb muscle masses respectively.

The AWGS SMI cut-off value to evaluate the loss of muscle mass in the diagnosis of sarcopenia was 5.7 kg/m^2^ in females (using the BIA method) [9,21]. Similarly, a study in which SMI was examined by the BIA method for Japanese women aged 18–39 years, the cut-off value was found to be 5.8 kg/m^2^ [22]. Furthermore, based on the results of a study on 1368 women aged 18–40 years [23], this value in the annual report of NHNS-J [19], published in 2017, was 5.7 kg/m^2^. Although the cut-off value varies by about 0.1 kg/m^2^ depending on the study, a cut-off value of 5.7 kg/m^2^ was adopted in this study based on reports of AWGS [9,21] and NHNS-J [19]. Therefore, SMI < 5.7 kg/m^2^ was considered to ‘correspond to the SMI cut-off value’ in this study. On the other hand, Janssen et al. [2] evaluated the muscle mass to diagnose sarcopenia and divided the values of SMI into 2 classes: ‘class 1’ values of SMI fell within 1–2 standard deviations below the young population's SMI average, and ‘class 2’ values were even lower than 2 standard deviations below the young population's SMI average (ie, same as the concept of SMI cut-off value). Based on the previous studies, which had measured SMI in Japanese females (6.8 ± 0.5 kg/m^2^ for each report [22,23]), a value equivalent to class 1, ie, 5.7 ≤ SMI < 6.3 kg/m^2^ was determined to be as ‘tending to correspond to the SMI cut-off value’ in the present study. In addition, individuals with ‘corresponding’ or ‘tended to correspond’ to the SMI cut-off value were evaluated as having a ‘low SMI’.

### 2.5 Statistical analysis

PAL classifications were defined as low-PAL (PAL < 1.6), moderate-PAL (1.6 ≤ PAL < 1.9), and high-PAL (1.9 ≤ PAL < 2.2). These classifications were based on IOM (low active; 1.4 ≤ PAL < 1.6, active; 1.6 ≤ PAL < 1.9, and very active; 1.9 ≤ PAL < 2.5) [15] or DRIs-J (low or level-I; 1.4 ≤ PAL < 1.6, moderate or level-II; 1.6 ≤ PAL < 1.9, and high or level-III; 1.9 ≤ PAL < 2.2) [16].

The chi-square (X^2^) test was used to compare the proportions of participants with low SMI in each PAL group. A residual analysis was conducted when a significant difference was observed. Means and standard deviations for height, weight, percentage of body fat, BMI, upper limb muscle mass, lower limb muscle mass, AMM, SMI, and sedentary time were calculated for each PAL group. One-way analysis of variance was used to compare the difference in means among the 3 groups, and multiple comparisons were performed using Tukey's test. In addition to signiﬁcance testing, effect sizes were calculated using Cohen's procedure (*d*).

These analyses were performed using JSTAT free software (ver. 12.5, Japan), and js-STAR (ver. 9.8.6j, Japan) free software. The level of statistical signiﬁcance was set at ≤ 5%. The effect size was established as small for *d* < 0.2, moderate for 0.2 ≤ *d* < 0.8, and large for *d* ≥ 0.8.

## 3 Results

**Fig. 1:**
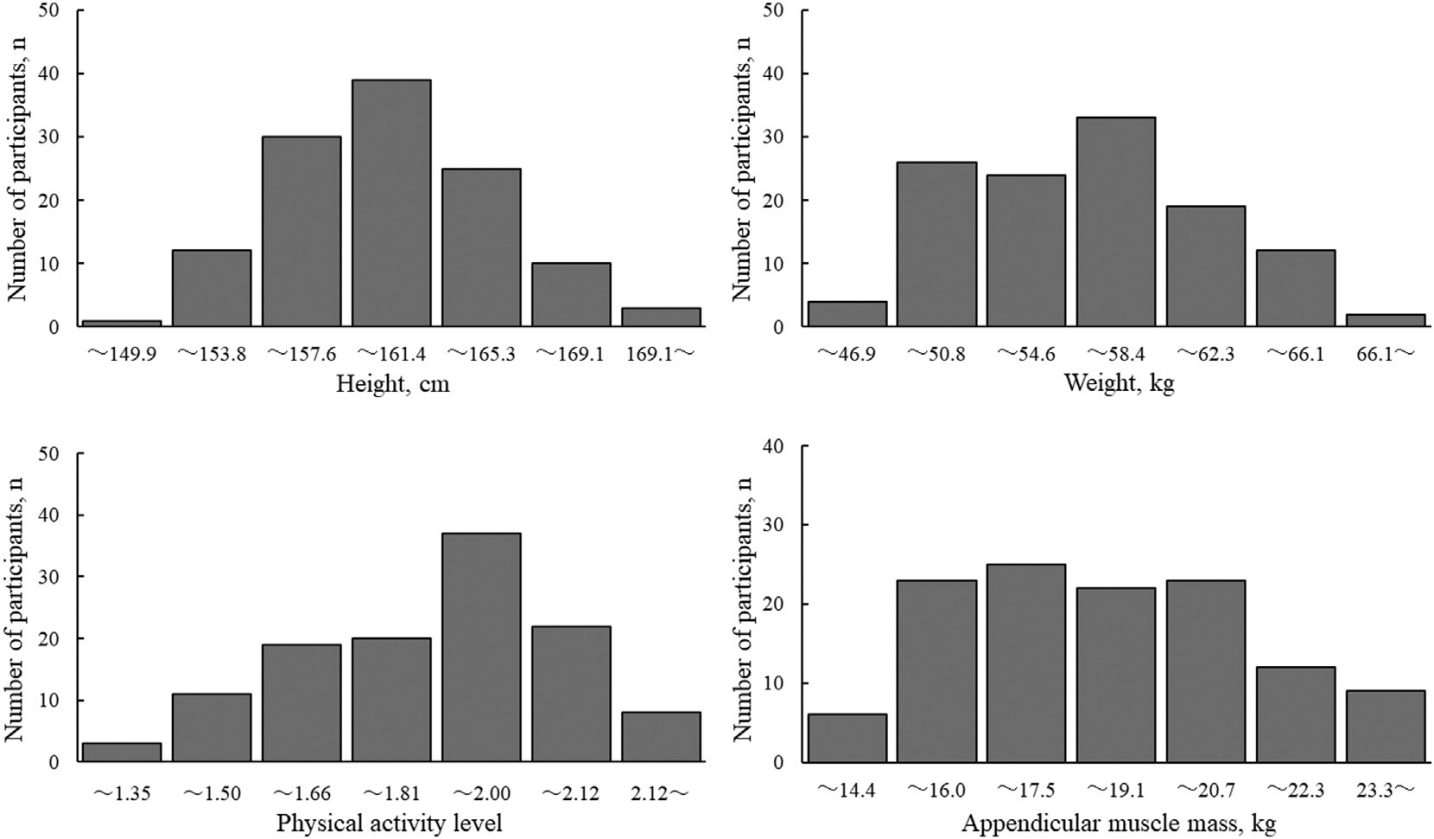
Frequency distribution of physical characteristics and physical activity level.

Table 1 shows the means and standard deviations of all participants for each variable. Fig. 1 shows the frequency distribution of the physical characteristics (height, weight, and AMM) and PAL. Of all the participants, 4 participants (3.3%) corresponded to the SMI cut-off value, and 28 participants (23.3%) tended to correspond to the SMI cut-off value. The distribution of the participants according to PAL was as follows: low-PAL 24, moderate-PAL 51, and high-PAL, 45. A total of 54.2% of the participants with low-PAL, 21.6% with moderate-PAL, and 17.8% with high-PAL had a low SMI. This result indicates that more than half of the female students, with an exercise habituation in the past, corresponded or tended to correspond to the SMI cut-off value if their current PAL was low (PAL < 1.6).

**Table 1:** Baseline characteristics of participants.

Number of participants, n		120		
Age, yr		19.0	±	0.8
Physical activity level		1.8	±	0.2
Sedentary time, h		7.9	±	2.2
Height, cm		159.5	±	4.8
Weight, kg		55.3	±	5.3
Body fat, %		24.9	±	5.0
Body mass index, kg/m^2^		21.7	±	1.8
Skeletal muscle mass index, kg/m^2^		7.12	±	0.93
SMI <5.7 kg/m^2^, n,%		4, 3.3%		
5.7 ≤ SMI <6.3 kg/m^2^, n, %)		28, 23.3%		
Muscle mass	Upper limb, kg	3.4	±	0.4
	Lower limb, kg	14.7	±	2.2
	Appendicular, kg	18.1	±	2.6

Values are mean ± standard deviation. SMI, skeletal muscle mass index.

Table 2 shows means and standard deviations for each variable among the three PAL groups. Sedentary time was shorter as PAL increased (9.9 ± 2.3 h for low-PAL, 8.4 ± 1.9 h for moderate-PAL, 6.4 ± 1.5 h for high-PAL). Significant difference was observed between the sedentary times of the 3 groups, and these effect sizes were large. SMI was significantly lower in the individuals with low-PAL (6.59 ± 0.89 kg/m^2^) than those with moderate-PAL (7.25 ± 0.91 kg/m^2^) or high-PAL (7.25 ± 0.86 kg/m^2^), and these effect sizes were also large. Upper limb muscle mass was significantly lower in the participants with low-PAL (3.2 ± 0.3 kg) than in those with high-PAL (3.5 ± 0.4 kg), and the effect size was moderate. Lower limb muscle mass was significantly lower in the individuals with low-PAL (13.4 ± 1.7 kg) than in those with moderate-PAL (14.9 ± 2.3 kg) and high-PAL (15.3 ± 2.2 kg), and the effect size between low-PAL and high-PAL was large, but was moderate between low-PAL and moderate-PAL. AMM was significantly lower in the students with low-PAL (16.6 ± 1.9 kg) than those with moderate-PAL (18.2 ± 2.6 kg) and high-PAL (18.8 ± 2.5 kg), and the effect size between low-PAL and high-PAL was large, but was moderate between low-PAL and high-PAL. There were no significant differences in the other variables based on PAL groups. These results indicate that (1) the sedentary time became shorter as PAL increased, (2) SMI of the individuals with low-PAL was lower than that of the individuals with moderate-PAL or high-PAL, (3) AMM was lower for the individuals with a decreased PAL especially for the lower limb muscle mass.

**Table 2:** Variables among the three physical activity level groups.

		Physical activity level											effect size (Cohen's *d*)		
		Low (I) (n = 24)			Moderate (II) (n = 51)				High (III) (n = 45)				I vs. II	I vs. III	II vs. III
		PAL = 1.5 ± 0.1			PAL = 1.8 ± 0.1				PAL = 2.0 ± 0.1						
Age, yr		19.1	±	0.7	18.9	±	0.7		19.0	±	0.9		0.2	0.1	0.1
Sedentary time, h∗∗		9.9	±	2.3	8.4	±	1.9	†	6.4	±	1.5	†^,^#	0.8	1.9	1.1
Body mass index, kg/m^2^		21.6	±	1.7	22.0	±	1.9		21.6	±	1.7		0.2	<0.1	0.2
Body fat, %		25.6	±	4.5	25.1	±	4.9		24.3	±	5.3		0.1	0.3	0.2
Skeletal muscle mass index, kg/m^2^∗∗		6.59	±	0.89	7.25	±	0.91	†	7.25	±	0.86	†	0.7	0.8	<0.1
Muscle mass	Upper limb, kg∗	3.2	±	0.3	3.4	±	0.4		3.5	±	0.4	†	0.4	0.8	0.3
	Lower limb, kg∗∗	13.4	±	1.7	14.9	±	2.3	†	15.3	±	2.2	†	0.7	0.9	0.2
	Appendicular, kg∗∗	16.6	±	1.9	18.2	±	2.6	†	18.8	±	2.5	†	0.7	1.0	0.2

Values are mean ± standard deviation.

∗ Denotes P < 0.05.

∗∗ Denotes P < 0.01.

† Denotes P < 0.05 vs. Low (I).

# Denotes P < 0.05 vs. Moderate (II).

## 4 Discussion

This study investigated the association between current PAL and SMI in female university students who had an exercise habit between the ages of 12 and 18 years (junior school to high school). The results showed that SMI was significantly lower for individuals with currently low-PAL than those with moderate-PAL or higher. In particular, the differences in muscle mass were larger for the lower limbs. More than 50% of the participants with currently low-PAL corresponded or tended to correspond to the SMI cut-off value.

The mean SMI in the present study was 7.12 kg/m^2^, which was higher than that calculated in previous studies using the BIA method. Two studies had reported an average of 6.8 kg/m^2^, one was conducted for 881 Japanese females aged 18–39 years [22] and the other was conducted for 1368 Japanese females aged 18–40 years [23]. A more recent study reported an average of 6.9 kg/m^2^ conducted for 154 Japanese females aged 18–40 years [24]. The higher SMI reported in the present study is thought to be due to the participants' past exercise habituation. However, the SMI in the present study was significantly lower in individuals with low-PAL than in those with moderate-PAL or higher. This suggests that in addition to PA in the past, maintaining current PAL at moderate in DRIs-J's classification [16] (active at IOM's classification [15]) or higher level is important for preserving and improving muscle mass. On the other hand, sedentary time decreased as PAL increased in the participants. Since sedentary time in the present study was extracted from the survey that was also used to calculate PAL, sedentary time becoming shorter as PAL increases may be a natural result. However, previous studies indicate that large amounts of sedentary time accelerate the loss of skeletal muscle mass in older adults [25]. Furthermore, a previous systematic review reported that a sedentary time of 9.5 or more hours daily significantly increases the risk of mortality [26]. A daily sedentary time of 9.5 or more hours was only present in the individuals with low-PAL in this study. The present study hence, suggests that acquiring the habit of not only maintaining the current PAL, but also minimizing the amount of sedentary time is important for improving the future health status by preventing muscle loss in the female students who used to exercise in the past.

Focusing on AMM, the upper limb muscle mass was significantly higher in individuals with high-PAL than in those with low-PAL, but the effect size was moderate. In contrast, the lower limb muscle mass was significantly higher in individuals with moderate-PAL or higher than in those with low-PAL, and the effect size between high-PAL and low-PAL was large. This indicates that the difference in muscle mass due to PA is larger in the lower limbs than in the upper limbs. Although ethnic differences exist in the age-associated changes in muscle mass [27], previous studies investigating age-related changes in muscle mass in Japanese people (same ethnic group as in the present study) highlight the importance of lower limb muscle mass [6,7]. They indicate that although the upper limb muscles show little change with advancing age, the decrease in the lower limb muscle mass for Japanese individuals begins in their twenties, and the reduction in the lower limb muscle mass with aging is the greatest among all the body parts [6,7]. Furthermore, normal weight obesity (NWO), which means that the body weight is normal; however, the body fat is high, which is considered an issue of body composition. NWO individuals were associated with a significantly lower AMM when compared with non-obese individuals in their thirties [28]. However, such a significantly lower AMM in individuals with NWO in their twenties and aged 18–19 years was observed only in the lower limbs [28]. Thus, it is suggested that the decrease in muscle mass, associated with the problems of body composition, is observed earlier in the lower limbs than in the upper limbs. Previous studies also reported that gait speed was significantly positively correlated with SMI among young women [10], and it was a predictor of NWO among participants aged 20–29 [28]. Therefore, the current PAL among female students especially affects the lower limb muscle mass, and it may cause difficulty in independent mobility in the younger generation, even in those who had an exercise habituation in the past.

In the annual report of NHNS-J [19], published in 2017, observed that 1.1% of women aged 60–64 years, 4.2% aged 65–74 years, and 14.4% aged 75 years and above corresponded to the SMI cut-off value. However, a systematic review on sarcopenia prevalence observed that 5.9%–40.3% of elderly women corresponded to the SMI cut-off value, while 60.3% had tended to correspond to the SMI cut-off value [29]. Thus, the prevalence of sarcopenia varies widely as mentioned in different reports, indicating maybe the involvement of various factors. In the present study, approximately 20% of the participants corresponded or tended to correspond to the SMI cut-off value, even if their PAL was moderate or high. If the nutritional intake of proteins is not optimal, exercise training cannot lead to a greater increase in muscle mass [30,31]. Changes in the behaviour of college students are observed not only at the level of PA status but also in the dietary patterns. Studies indicate that when compared to the level at college entry, caloric intake in female students decreased significantly at 5 months [13] or 12 months [32]. Furthermore, 1 study that investigated the changes in several behaviors and body weight in males and females from the final year of high school until the second year of college/university reported that a decrease in the fruit/vegetable intake contributed to weight change in females only [33]. Another study conducted on Japanese female students reported that individuals who skipped meal(s) and had insufficient PA had markedly lower SMI [11]. Therefore, future studies should consider dietary status as well.

Several limitations associated with the present study warrant mention. First, the sample size was relatively small. Although this study focused on female students who had an exercise habit in the past, the number of overall young Japanese women with exercise habits is low [19]. Therefore, the sample size was small if it was limited to those who had a habit of exercising among the participants in this study. Second, other factors related to muscle mass should also be considered. As mentioned above, muscle mass is related not only to PA but also to nutritional intake, especially protein intake; therefore, dietary status should be considered in future studies. Further, the pre-set study only investigated having past sports experience during junior and high school. Details of PA during this period, including for sedentary individuals, should also have been investigated accordingly. In addition, only past exercise habituation was examined in this study, and the SMI at that time was unclear. Therefore, a longitudinal SMI study is necessary. Third, measurement methods also need to be considered in the study. Although PAL was evaluated using a factorial method, future studies should also consider measuring the actual amount of PA. Furthermore, although the accuracy of measuring body composition using dual- or multi-frequency BIA method is relatively high, it is necessary to use more accurate methods such as dual-energy X-ray absorptiometry (DXA) for the determination of muscle mass. However, the DXA method should consider the influence of exposure to X-rays. Finally, sarcopenia diagnostic criteria include evaluation of muscle strength and physical performance, in addition to the evaluation of muscle mass. Although this study focused on muscle mass based on several reasons mentioned in the introduction section, the evaluation of muscle strength or physical performance is also important. Further, it is a prerequisite that sarcopenia is diagnosed at the age of 65 years or older. However, this study showed that even young females between the ages of 18 and 21 years, depending on their PAL, corresponded to the SMI cut-off value in the diagnosis of sarcopenia. Furthermore, the present study suggests that a moderate current PAL in the DRIs-J classification [16] (active at IOM's classification [15]) or higher PAL even for young females who used to exercise in the past is required in order to maintain muscle mass, especiall in the lower limbs.

## 5 Conclusions

The present study investigated the association between current PAL and SMI in female university students with exercise habituation in the past during junior and high school. Individuals with currently low-PAL had a significantly lower SMI, especially for lower limb muscle mass, than those with moderate-PAL or higher. Among the participants with currently low-PAL, more than 50% corresponded or tended to correspond to the SMI cut-off value for sarcopenia diagnosis. Therefore, more than half of the female students' with currently low-PAL, even those who used to exercise in the past, corresponded to the cut-off value for muscle mass loss in sarcopenia diagnosis. Furthermore, this muscle loss was particularly greater in the lower limbs. These results suggest that it is important to maintain a moderate current PAL in the DRIs-J classification [16] (active at IOM's classification [15]) or higher PAL, even in young women with an exercise habituation in the past, to maintain SMI.

## CRediT author statement

**Kazushige Oshita**: Conceptualization, Investigation, Data curation, Writing - original draft, Writing - review & editing. **Ryota Myotsuzono**: Investigation, Data curation, Writing - review & editing.

## Conflicts of interest

The authors declare no competing interests.
